# Making Connections: Mesenchymal Stem Cells Manifold Ways to Interact with Neurons

**DOI:** 10.3390/ijms23105791

**Published:** 2022-05-21

**Authors:** Olga Tarasiuk, Elisa Ballarini, Elisabetta Donzelli, Virginia Rodriguez-Menendez, Mario Bossi, Guido Cavaletti, Arianna Scuteri

**Affiliations:** Experimental Neurology Unit and Milan Center for Neuroscience, School of Medicine and Surgery, Milano-Bicocca University, 20900 Monza, Italy; olga.tarasiuk@unimib.it (O.T.); elisa.ballarini@unimib.it (E.B.); elisabetta.donzelli@unimib.it (E.D.); virginia.rodriguez1@unimib.it (V.R.-M.); mario.bossi@unimib.it (M.B.); guido.cavaletti@unimib.it (G.C.)

**Keywords:** mesenchymal cells, neurons, tunneling nanotubes, gap junction, extracellular vesicles

## Abstract

Mesenchymal Stem Cells (MSCs) are adult multipotent cells able to increase sensory neuron survival: direct co-culture of MSCs with neurons is pivotal to observe a neuronal survival increase. Despite the identification of some mechanisms of action, little is known about how MSCs physically interact with neurons. The aim of this paper was to investigate and characterize the main mechanisms of interaction between MSCs and neurons. Morphological analysis showed the presence of gap junctions and tunneling nanotubes between MSCs and neurons only in direct co-cultures. Using a diffusible dye, we observed a flow from MSCs to neurons and further analysis demonstrated that MSCs donated mitochondria to neurons. Treatment of co-cultures with the gap junction blocker Carbenoxolone decreased neuronal survival, thus demonstrating the importance of gap junctions and, more in general, of cell communication for the MSC positive effect. We also investigated the role of extracellular vesicles; administration of direct co-cultures-derived vesicles was able to increase neuronal survival. In conclusion, our study demonstrates the presence and the importance of multiple routes of communication between MSCs and neurons. Such knowledge will allow a better understanding of the potential of MSCs and how to maximize their positive effect, with the final aim to provide the best protective treatment.

## 1. Introduction

Mesenchymal Stem Cells (MSCs) are adult stem cells well known by now in every field of regenerative medicine, from cardiology to immunology and neurology [1]. They are considered an unconventional tool to fight against several different diseases, even if characterized by various clinical manifestations and overall by assorted etiopathogenetic mechanisms. With a special focus on neurodegenerative diseases, such an intriguing role mainly resides on the manifold properties of MSCs, ranging from immunomodulatory and anti-inflammatory effects to a pro-survival action [2]. Nevertheless, the ability to be seemingly effective for the treatment of such diverse neurological diseases has raised questions about the effective mechanisms of MSC action, whose knowledge is essential for actually considering a clinical use of MSCs. So far, several hypotheses have been made with the most intriguing one being the MSC differentiation to replace damaged cells [1]. Recently, other assumptions have become more convincing, such as the release of trophic factors, as well as the direct support to cellular survival. It is believed that stem cells can secrete some neuronal factors such as BDNF, GDNF and NGF that promote neurite outgrowth and neural function recovery [3]. In fact, several studies have shown promising results of MSC use in in vitro models of neurodegenerative diseases, such as Parkinson’s Disease and Alzheimer’s Disease (AD), where it has been demonstrated that MSCs, as well as their secretome, can interfere with misfolded TAU proteins, thus promoting survival and increasing metabolic activity in AD in vitro models [4]. In the same way, in in vivo models, intracerebral injected MSCs were able to reduce accumulation of amyloid-β (Aβ), to decrease hyperphosphorylated TAU protein levels [5,6] and to improve learning and memory abilities [7]. In other studies, it has been demonstrated that MSCs directly co-cultured with sensory neurons were able to strongly increase neuronal survival and to protect neurons from different toxic stimuli [8,9]. In such a model, direct contact between MSCs and neurons was pivotal to achieve the protection, whilst a less powerful effect was obtained by soluble factors. In addition, it is more and more evident that MSCs possess the ability to change their expression pattern according to the surrounding milieu, thus properly responding to the exogenous stimulation [10]. Starting from these observations, we focused our investigation on the characterization of the key mechanisms of interaction between MSCs and neurons.

Intercellular communications are crucial for all biological processes, such as the maintenance of tissue homeostasis, regulation of normal cellular function and response to external environmental signals that affect cell survival [11]. Among the different mediators of intercellular interactions, an important role is carried out by gap junctions [12].

Gap junctions are clusters of intercellular channels composed of “connexins” that create a direct connection between the cytoplasm of two neighboring cells for exchange of nutrients and large molecules between the cells [13]. MSCs have been reported to form gap junctions with different cells, such as bronchial epithelial cells [14], epithelial cells [15], and overall cardiomyocytes, with important effects on cardiac repair [16,17].

Another impressive means of communication that allows long-distance cell-to-cell contact is represented by the relatively recently reported Tunneling Nanotubes (TNTs). TNTs have been described as very small caliber cytoplasmic bridges connecting cells and allowing the exchange of subcellular structures. In particular, TNTs are long tubular structures, which could be of two types: thin, with a diameter of 50–200 nm and rich in F-actin filaments, or thick, with a diameter between 600–700 nm, containing F-actin microfilaments and microtubules. They can span several tens to hundreds of microns, connecting two cells together. TNTs seem to be key structures in viral transmission [18], however, they have been recently described as important cross-talk mediators: they allow the cell-to-cell trafficking of various cellular components, including proteins, RNAs, and even organelles (in case of thick TNTs), such as mitochondria [19]. 

Moreover, it has been shown that MSCs secrete vesicles containing important molecules. Extracellular vesicles (EVs) are a heterogeneous group of biologically active membrane-encompassed vesicles released by cells and, according to their size and biogenesis, EVs can be divided into three main types: exosomes, microvesicles, and apoptotic bodies. EVs are found in numerous body fluids as well as in the medium of cultured cells and can carry lipids, proteins, enzymes, and coding and noncoding RNA molecules. EVs play an important role in crosstalk between cells and modulate target cell functions. Different studies confirm that MSC-derived EVs have regenerative and anti-inflammatory properties and improve post-stroke neuroregeneration [20], traumatic brain injury [21], and wound healing [22], although no unifying hypothesis exists for the underlying mechanism promoting neuronal growth and survival.

Here, we verified the presence and the importance of all these intercommunication structures for the positive effect of MSCs on neuronal survival. The demonstration of such a “dynamic interaction” between cells and its important role for neuronal survival will underline the ability of MSCs to properly react to the surrounding inputs from the other cells. Moreover, the knowledge of the interaction mechanisms between MSCs and neurons will allow one to maximize the positive MSC effect by introducing the most advisable changes, with the final aim to provide a protective treatment for neurons.

## 2. Results

### 2.1. Morphological Analysis of Intercellular Communication Structures

**Figure 1:**
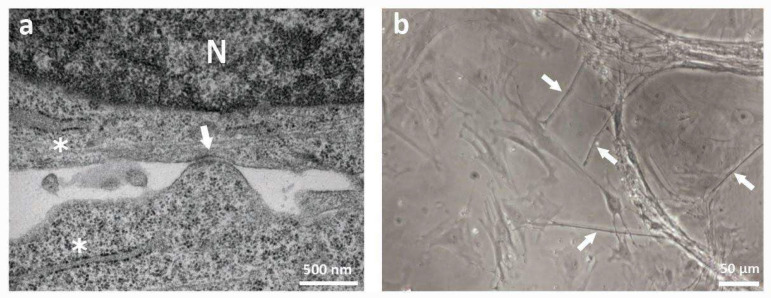
Morphological analysis of intercellular communication structures. (**a**) Electron microscopy analysis of direct co-cultures of neurons and MSCs after 30 days, showing the presence of desmosomal-like structures (white arrow). The * indicate cisternae of reticulum endoplasmic (RE). The black dots observed on the RE surface are ribosomes. The letter N indicates the nucleus. (**b**) Optical microscope analysis of direct co-cultures of neurons and MSCs after 30 days. White arrows indicate the presence of very thin structures connecting neuronal processes and MSCs.

As previously reported [8], the contact between MSCs and neurons leads to an important and prolonged increase of sensory neuronal survival, with direct interaction between MSCs and neurons reported to be pivotal. To deepen the knowledge of the mechanisms involved in such an interaction, the neurons, alone and in direct co-culture with MSCs, were analyzed at the electron microscope, which showed the presence of desmosomal-like structures only in the co-cultures (Figure 1a and Figure S1). Moreover, besides the presence of gap junctions, under the optic microscope in the direct co-cultures, it was possible to observe some very thin processes connecting MSCs to neurons (Figure 1b), resembling Tunneling Nanotubes (TNTs), the newly discovered form of intercellular communication.

**Figure 2:**
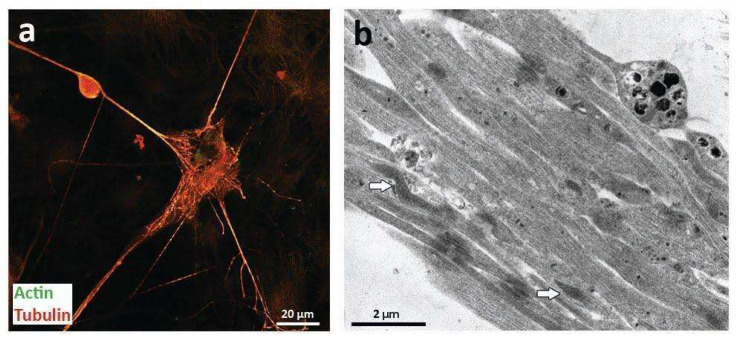
Morphological characterization of TNTs. (**a**) confocal analysis on direct co-cultures of neurons and MSCs after 30 days showing the presence of actin (green) and tubulin (red). The yellow spots show the co-localization of the proteins. (**b**) Electron microscopy analysis of TNTs found direct co-cultures of neurons and MSCs after 30 days, with a diameter consistent with that already reported for thick TNTs. White arrows indicate the presence of mitochondria.

To characterize these structures as TNTs, electron microscopy and immunofluorescence analysis was performed in the direct co-cultures. In particular, TNTs could be identified mainly by morphological criteria [23]: in the case of thick TNTs, a diameter of 600–700 nm and the presence of filaments of actin and tubulin. As shown in Figure 2 and Figure S1, the thin processes observed in direct co-cultures were positive for both criteria, thus being considered as TNTs (Figure 2).

### 2.2. Direct Interactions between MSCs and Neurons

The formation of TNTs and gap junction-like structures in the direct co-cultures let us hypothesize a direct and dynamic interaction between MSCs and neurons. In fact, these structures are known to potentially mediate the exchange of some molecules.

**Figure 3:**
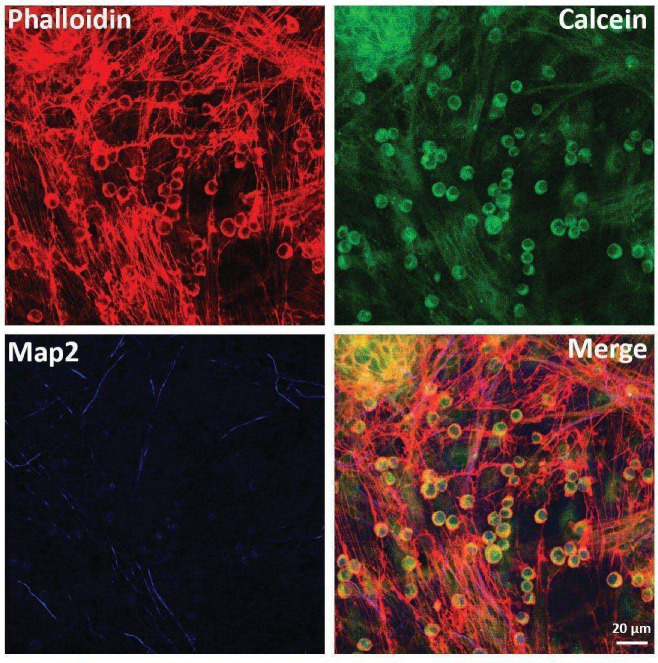
Analysis of the exchange of cytoplasmic materials. MSCs previously stained with the fluorescent diffusible vital dye Calcein were directly co-cultured with neurons. After 30 days of co-culture, immunofluorescence was performed to show the putative migration of Calcein. In red: Phalloidin; in green: cCalcein; in blue: Map2.

Once the presence of direct communication structures was proved, we moved to verify the potential exchange of cellular materials between cells. MSCs previously stained with the vital fluorescent cytoplasmic dye Calcein were added to sensory neurons for the co-culture set up. As shown in Figure 3, after 30 days it was possible to observe sensory neurons stained with Calcein, thus demonstrating a direct exchange of cytoplasmic materials from MSCs to neurons. On the contrary, when neurons were stained with Calcein before the co-culture set up, we did not observe any dye transfer to MSCs, thus demonstrating that only MSCs could “donate” cytoplasmic materials to neurons (data not shown).

**Figure 4:**
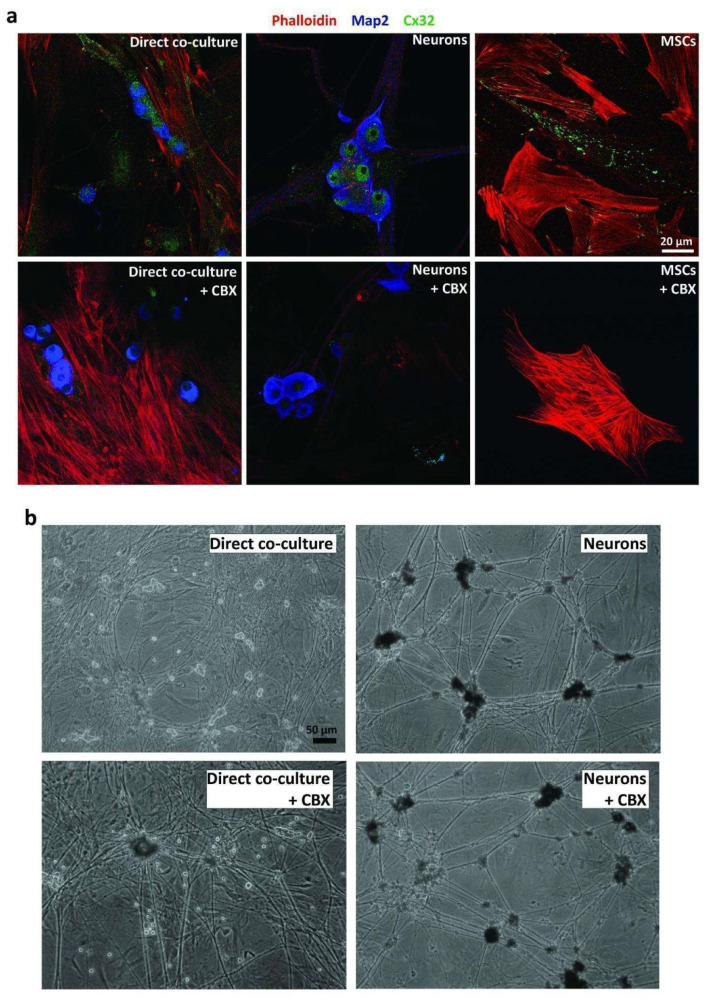
Analysis of the role of gap junctions. (**a**) Confocal analysis for the presence of Cx32 in direct co-cultures, neurons cultured alone and MSCs cultured alone after 30 days of culture, with and without the gap junction blocker Carbenoxolone (CBX). In red: Phalloidin; in green: Cx32; in blue: Map2. (**b**) Morphological analysis of neurons and direct co-cultures with or without the exposure to Carbenoxolone (CBX) for 30 days.

To confirm the importance of such a connection between MSCs and neurons, we used Carbenoxolone (CBX), a pan-gap junction blocker [24]. To check the effectiveness of CBX, we examined its effect on Connexin 32, largely represented in neurons. CBX treatment decreased the expression of Connexin 32 (Cx32) in all of the examined samples (Figure 4a), resulting in a reduction of neuronal survival in co-cultures, thus demonstrating the important role of gap junctions, and direct interactions in general, in the positive effect of MSCs (Figure 4b).

### 2.3. Mitochondrial Exchange

**Figure 5:**
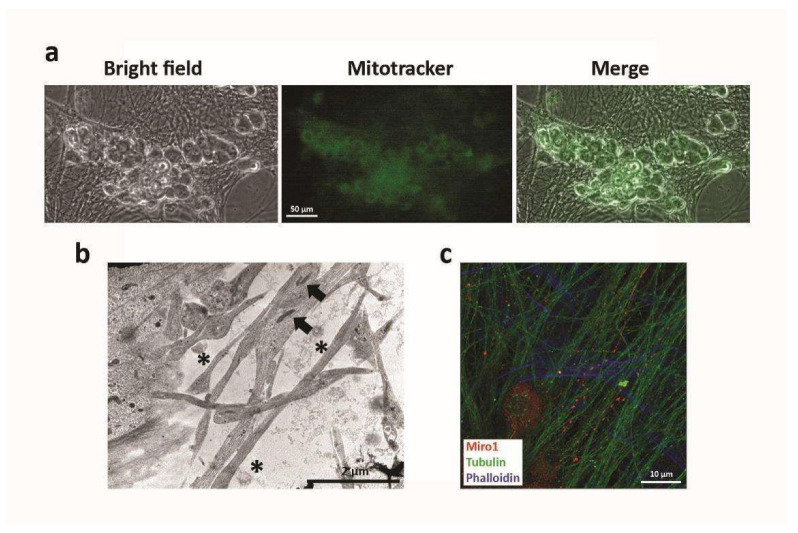
Analysis of mitochondrial exchange. (**a**) Evidence of the transfer of green Mitotracker from MSCs to neurons. MSCs previously stained with Mitotracker (green) were added to neurons for setting up direct co-cultures. By the time-lapse cell imaging system Biostation, both in fluorescence and in bright field, the flow of Mitotracker was analyzed and its presence inside neurons was shown. (**b**) Electron microscopy analysis of TNTs in direct co-cultures revealed the presence of mitochondria (black arrows). The * indicated the presence of EVs, probably exosomes according to their dimensions. (**c**) Confocal microscopy analysis of direct co-cultures for the presence of Miro1 along the processes. In green: Tubulin; in red: Miro1; in blue: Phalloidin.

After the demonstration of transfer of cytoplasmic materials from MSCs to neurons, we tried to identify the possible molecules “donated” to neurons. An intriguing hypothesis suggests that TNTs could mediate the exchange of mitochondria [25]. To verify such a hypothesis in our model, MSCs were previously stained with mitochondrial dye Mitotracker, and after that co-cultured with neurons. Then, by using a live-imaging system, we observed that mitochondria stained with Mitotracker passed from MSCs to neurons (Figure 5a). In addition, electron microscopy analysis demonstrated the presence of mitochondria along TNTs (Figure 5b) and immunofluorescence analysis demonstrated the presence of Miro1, a protein regulating mitochondrial movement along TNTs (Figure 5c).

### 2.4. Extracellular Vesicles Analysis

**Figure 6:**
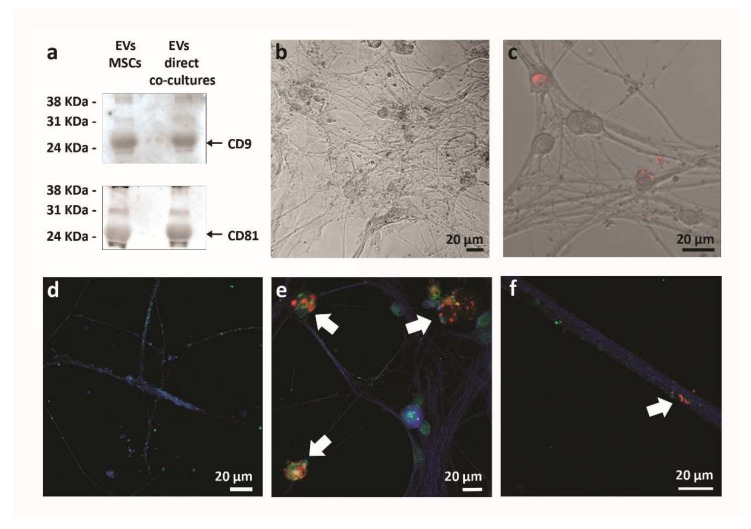
Analysis of EVs. (**a**) EVs isolated from MSCs cultured alone or EVs collected from direct co-cultures were analyzed by Western Blot for the presence of CD9 and CD81, markers of exosomes. (**b**) Neurons without the addition of EVs. (**c**) Neurons added with PKH26 stained EVs (red) from direct-co-cultures. Confocal analysis of (**d**) neurons without the addition of EVs, (**e**) neurons added with PHK26 stained-EVs (red) derived from direct-cultures and (**f**) neurons added with PHK26 stained-EVs (red) derived from MSCs. In green: CD9; in red: PKH26 stained EVs; in blue: Map2. White arrows show CD9 colocalizing with PHK26-stained EVs (yellow).

Lastly, the analysis of the co-cultures at the electron microscope also showed the presence of some vesicles “traveling” from MSCs to neurons (see Figure 5b). Due to their dimension (30–150 nm), these vesicles could be considered as exosomes, according to the literature criteria [26]. Since exosomes are considered short-distance mediators [27], we further investigated this interaction. We firstly collected and characterized the vesicles secreted in the culture medium by MSCs, both cultured alone (EVs-MSCs) and in direct co-culture with sensory neurons (EVs-direct co-cultures). In order to verify the presence of exosomes in EV preparations, we performed a Western Blot analysis to identify the proteins CD9 and CD81, which are considered exosomes markers. As shown in Figure 6a, both EVs-MSCs and EVs-direct co-cultures displayed the bands corresponding to CD9 and CD81 proteins, thus confirming the presence of exosomes in both the EV preparations.

EVs isolated from the culture medium of MSCs cultured alone or in direct co-culture with neurons, were stained with the lipophilic membrane dye PKH26 and added once a week to neurons cultured alone, up to 6 weeks. As shown in Figure 6, neurons were able to internalize stained EVs (Figure 6c) that co-localized with exosomal marker CD9 and were distributed mostly in neuronal cell bodies and along neuronal processes (Figure 6e,f). Neurons cultured alone (Figure 6d) not receiving EVs represent a negative control and did not show any staining.

**Figure 7:**
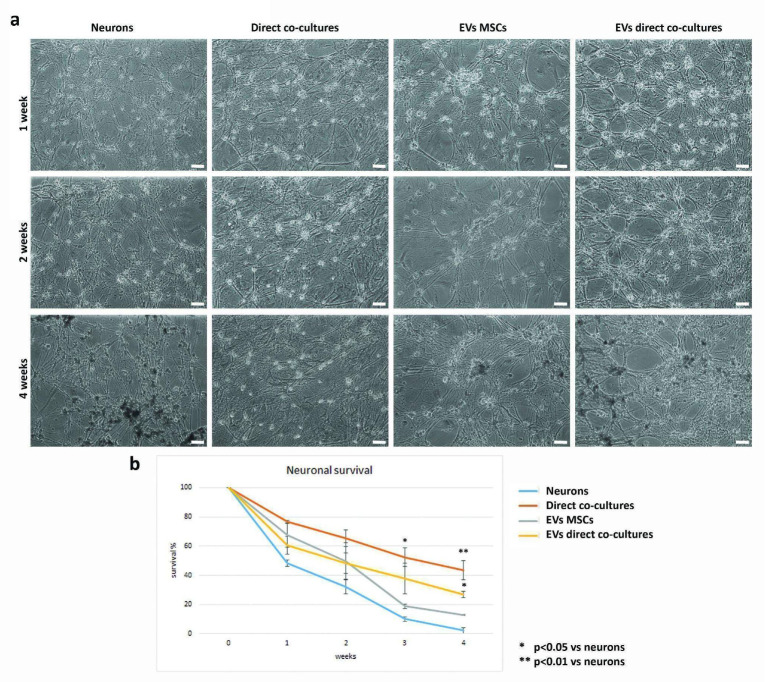
Evaluation of neuronal survival after the addition of EVs. (**a**) Morphological analysis of the effect of EVs, derived from MSCs or from direct co-cultures, on neuronal survival at different time points (1, 2, and 4 weeks of culture), with respect to neurons cultured alone (Neurons) or in direct co-cultures with MSCs. Bar = 50 µm. (**b**) Graph of the effect of EVs, derived from MSCs or from direct co-cultures, on neuronal survival at different time points (1, 2, 3 and 4 weeks of culture), compared to neurons cultured alone (Neurons) or in direct co-culture with MSCs.

To investigate EVs’ role in MSC-mediated increase of neuronal survival, sensory neurons were incubated with EVs-MSCs or with EVs-direct co-cultures. Light microscopy showed that neurons cultured alone, without the addition of EVs, showed a suffering appearance starting from 1 week of culture, with evident signs of neurite degeneration and neuronal death after 4–6 weeks of culture, as shown in Figure 7a. Neurons in direct co-culture with MSCs had a healthier appearance and they were still alive after 4–6 weeks. Neurons receiving EVs collected from MSC medium had a slightly better survival with respect to control neurons, although the difference was not statistically significant. On the contrary, neurons receiving EVs isolated from direct co-cultures were able to survive longer than control neurons (Figure 7b).

## 3. Discussion

Neuronal cells possess a very limited regenerative capacity and, for this reason, finding a mechanism to support their survival can represent significant progress towards the treatment of different neurodegenerative diseases. A new hope that is gaining ground is cell-based therapy, a promising approach with encouraging results in different models of nervous system diseases [28]. In such a therapeutic context, MSCs play a leading role, being able to stimulate regeneration, neuronal survival, and immunomodulatory properties, together with a high biosafety profile. So far, many mechanisms of action have been hypothesized, from the release of trophic factors to direct interactions [29], thus suggesting that MSCs could exploit more than one mechanism, tuning their action according to the specific framework. To do that, it is fundamental that MSCs could dynamically interact with the surrounding cells. Here, we unraveled different ways by which MSCs talk to neurons, also demonstrating that such interactions are important for the MSC-dependent support to neuronal survival.

In particular, we demonstrated the formation of three different intercommunication structures between MSCs and neurons: the already described gap junctions, the more newly discovered TNTs, and the short-distance vesicles traffic. Probably through all these structures, we observed the transfer of cytoplasmic materials from MSCs to neurons, but not vice versa. Among the materials donated by MSCs, we identified the presence of mitochondria, which could be a fundamental mediator of neuronal survival increase [30].

All these mechanisms have been already reported separately in studies on MSCs and other cells, in some case also with neurons, but this is the first time that all the three mechanisms are demonstrated in the same culture involving MSCs and neuronal cells, overall with a relation to MSC-dependent neuronal survival increase.

In fact, the formation of gap junctions has been already demonstrated to be pivotal between neurons and astrocytes, for neuron survival and neurite elongation [31]. Such a mechanism has been considered as a “passage structure” by which astrocytes could modulate neurons [32,33]. In the same way, neurons seem to be able to also exploit gap junctions to act on MSC differentiation toward a neural phenotype [34]. In our model we demonstrated the influence, by gap junctions, of MSCs on neurons, rather than vice versa; in fact, blocking these structures overrides the pro-survival effect observed in direct co-culture of neurons with MSCs. This result is in line with other publications where exchange of cytoplasmic and mitochondrial transfer between MSCs and lung epithelial cells [35] was described.

Concerning TNTs, their formation between MSCs and neurons has not yet been reported, while TNTs have already been described as heterotypic bridges in the co-cultures of MSCs with other cell types, such as cardiomyocytes, epithelial cells, macrophages, renal tubular cells and cancer cells [19], as very rapid communication structures [35], but also as a spreading mean for pathogens [36] or pathological structures, such as α-synuclein [37]. In our study, we also showed the formation of TNTs between MSCs and neurons, thus reinforcing the hypothesis, suggested by other authors, of a mitochondria transfer through TNTs, already reported between MSCs and different cellular types such as cardiomyocytes [38] and hepatocytes [39]. Mitochondria are involved in many physiological processes, as well as in pathological states [40]. The ability of MSCs to “donate” healthy mitochondria, already demonstrated with cardiomyocytes [16,41], could actually represent a valid mechanism for neuronal protection: mitochondrial dysfunction is a key element of neuronal degeneration. By donating healthy mitochondria, MSCs could prevent neuronal degeneration, thus improving survival.

In the literature, it has been hypothesized that some environmental stress factors could be necessary to trigger TNT development between cells. Wang et al. [42] demonstrated TNT formation between astrocytes and neurons, suggesting that the stressed cells promote TNT development, which extends to the unstressed cells. In our model, neurons suffered more and more during the culture, which could be the stress situation triggering TNT formation, being the further demonstration of a dynamic interaction between MSCs and neurons, despite the observation that only MSCs could “donate” molecules to neurons. Some studies suggest that directionality of mitochondrial transfer could be determined by some stress signals produced by the suffering cells [43], like damaged mitochondria, released mtDNA or mitochondrial products [44,45,46], thus triggering a signal for mitochondrial transfer, although the mechanism of such an exchange is a complex micro environmental process and it remains to be further elucidated.

Besides TNTs and gap junctions, we also demonstrated that EVs could be engrafted by neuronal cells, and in particular those from direct co-cultures showed a positive effect on neuronal survival. The importance of the secretome of MSCs and EVs has already been demonstrated in several models such as acute kidney injury [47], ischemia [48], Alzheimer’s disease [49], and osteoarthritis [50], ascribing this beneficial effect to the transfer of specific mRNAs and growth factors, able to modulate cellular processes such as apoptosis, cell proliferation, angiogenesis and inflammation [47,51].

One of the most significant findings of the present paper is the observation that EVs derived from the direct co-culture are more effective at supporting neuronal survival than those spontaneously released by MSCs alone. Other studies have confirmed the possibility to “prime” MSCs in order to strengthen their effect [52,53,54,55,56]. A common means to “prime” MSCs is their exposure to hypoxic conditions, as well as to some neurotrophic factors or neuroactive molecules before their use in different cellular models; in this way, MSCs could improve their supporting abilities [52,53,54,55,56]. In our model, the direct co-culture with neurons could be considered as a “priming” conditioning method for MSCs; in some ways, neurons signal MSCs about their state, and MSCs consequently adapt their secretome. It is likely that MSCs do not spontaneously express or release the pivotal molecules, but rather they are firstly affected by the surrounding milieu, and only as a response they express or release specific factors. By this view, MSCs possess the ability to properly react to the surrounding inputs from the other cells, in this case neurons. To do that, the establishment of a “dynamic interaction” among these cells is fundamental.

In conclusion, we can affirm that the present paper provides a further insight into MSC action: the observed positive effect of MSCs on neuron survival when in co-culture likely involved not just a single mechanism, but at least three different interaction strategies, ending up with the donation of cytoplasmic material, in particular mitochondria, which can alleviate neuronal stress.

## 4. Materials and Methods

The study was approved by the Milano-Bicocca University ethics committee (N 0035828/13) and it was performed in conformity with the institutional guidelines, in compliance with national (DL n. 26/2014) and international (EEC Council Directive 2010/63/EU, OJL 358, Dec. 1987; NIH Guide for the Care and Use of Laboratory Animals, US NRC, 1996) laws and policies and with the ARRIVE guidelines.

### 4.1. DRG Neurons Primary Cultures

DRG from 15-day old Sprague-Dawley rat embryos (Envigo, Casatenovo, Italy) were removed and dissociated with trypsin. Then neurons were cultured on collagen-coated dishes for 5 days in AN2 medium composed of MEM (Euroclone S.p.A., Pero, Italy) and 15% calf bovine serum, 50 mg/mL ascorbic acid (Sigma Chemical Co., St. Louis, MO, USA), 1.4 mM glutamine (Euroclone), 0.6% glucose (Sigma) supplemented with 5 ng/mL NGF (Euroclone) and 10^−5^ M Fudr (Sigma) to remove satellite cells. Neurons were then incubated with AN2 medium with 5 ng/mL NGF. The AN2 medium was changed twice a week.

### 4.2. MSCs Cultures

MSCs were obtained from the bone marrow of Sprague-Dawley rats (Envigo) by flushing the femur and tibia diaphysis with 2 mL/bone of alpha MEM with 2 mM Glutamine and antibiotics (100 U/mL Penicillin G and 100 µg/mL Streptomycin Sulfate) (Euroclone). MSCs were cultured in a humidified incubator at 37 °C with 5% CO_2_ in alpha-MEM medium plus 2 mM Glutamine, antibiotics and 20% fetal bovine serum (Euroclone).

### 4.3. Co-Cultures

MSCs (passage 4–7) were trypsinized and added on neurons at a density of 10^4^ cells/cm^2^, co-cultures were maintained in AN2 + 5 ng/mL NGF. Carbenoxolone (CBX, Sigma) was dissolved in water and then added to the culture medium at the concentration of 100 μM.

### 4.4. Calcein and Mitotracker

The cytoplasmic fluorescent dyes Calcein (Becton Dickinson Italia S.p.A., Milan, Italy) and Mitotracker (Thermo Fisher Scientific, Monza, Italy) were added to MSC culture medium at a concentration respectively of 4 µM for 30 min and 200 nM for 45 min at 37 °C. Then, the medium with dyes was removed, the MSCs cultures were washed with PBS (Euroclone), trypsinized and added to neuronal cultures at a concentration of 10^4^ cells/cm^2^. The Time Lapse Imaging Biostation system (Nikon Instruments Inc, Amsterdam, The Netherlands) was used to obtain live detection of Calcein or Mitotracker flow.

### 4.5. Immunofluorescence and Electron Microscope Analysis

The cultures were washed with PBS and then fixed in 4% paraformaldehyde. Immunostaining was performed using anti-Map2 (1:50, Chemicon Int., Temecula, CA, USA), Actin (1:50, Santa Cruz Biotechnology, Dallas, TX, USA), Tubulin (1:200, Sigma), and Miro1 (1:50, GeneTex, Irvine, CA, USA) as primary antibodies. Phalloidin (1:20, Rockland, Gilbertsville, PA, USA) was used to visualize MSCs. Anti-CD9 (1:50, Sigma) was used to stain exosomes to analyze its colocalization with PKH26 (Sigma). Anti-Cx32 (1:50, Abcam Ltd., Cambridge, MA, USA) was used to stain gap-junctions. Cells were then examined using confocal laser microscopy, carried out with a LSM 710 confocal microscope (Carl Zeiss, Milan, Italy).

For the electron microscope, co-cultures and neurons alone were fixed for 30 min in 4% paraformaldehyde (Sigma) and 2% glutaraldehyde (Sigma) in phosphate buffer 0.12 M, postfixed in 1% OsO_4_ in cacodylate buffer (Sigma) for 30 min, dehydrated with increasing concentration of ethanol and embedded in epoxy resin. Ultrathin sections (70 nm) were stained with uranyl acetate and lead citrate and they were observed with a Philips CM10 transmission electron microscope (Philips Medical Systems S.p.A., Monza, Italy).

### 4.6. EVs Isolation and Administration

Medium collected from MSCs cultured alone or from direct co-cultures of MSCs and neurons was collected once a week for three weeks. Extracellular vesicles were isolated from the culture media using ExoQuick-TC™ Exosome precipitation solution (System Bioscience, Palo Alto, CA, USA) according to manufacturer’s instructions, allowing precipitation of exosomes and microvesicles between 30 and 200 nm size. The pellet with the precipitated microvesicles was resuspended in 100 µL PBS. The protein concentration was verified by Bradford assay. Vesicles were then stained with lipophilic membrane red dye PKH26 according to manufacturer’s instructions (Sigma Chemical Co.). Tubes were centrifuged at 190,000× *g* for 2 h at 4 °C. The supernatant was aspirated and the pellet was resuspended in PBS to the final concentration of 10 ng/µL. Then, 25 µg of extracellular vesicles were added once a week, up to 6 weeks, to neurons plated in 10 cm^2^ dishes without MSCs added.

### 4.7. Immunoblotting

In order to identify the exosomal markers CD9 and CD81 in extracellular vesicle preparation, Western Blot was performed. Briefly, 50 µg of EVs in Laemmli buffer were separated by SDS-PAGE (13% acrylamide) and transferred to a nitrocellulose filter. Membranes were blocked with 5% non fat milk (for CD9 immunoblotting) or 5% BSA (for CD81 immunoblotting) and incubated with the primary antibodies anti-CD9 (1:2000, Sigma) or anti-CD81 (1:500, Thermo Fisher Scientific) for 1 h at RT. Afterwards, membranes were washed, incubated with peroxidase-conjugated secondary anti-rabbit antibody (1:2000, Perkin Elmer Italia, Milan, Italy) for 1 h at room temperature. Chemiluminescence signal was developed with a detection kit (LiteUP, Euroclone) according to manufacturer’s instructions and then acquired by Amersham ImageQuant800 western blot imaging system (Cytiva Europe GmbH, Buccinasco, Italy).

### 4.8. Survival Evaluation and Statistical Analysis

Neuronal survival was evaluated by counting the viable neurons, which were characterized by a birefringent outline that was absent in dead cells. Statistical analysis of the data was carried out using the one-way Anova test and Tukey post-test with the GraphPad Prism statistical package (GraphPad Software, San Diego, CA, USA).

## Supplementary Materials

The following are available online at https://www.mdpi.com/article/10.3390/ijms23105791/s1.

## References

[1] Han Y., Li X., Zhang Y., Chang F., Ding J. (2019). Mesenchymal Stem Cells for Regenerative Medicine. Cells.

[2] Weiss A.R.R., Dahlke M.H. (2019). Immunomodulation by Mesenchymal Stem Cells (MSCs): Mechanisms of Action of Living, Apoptotic, and Dead MSCs. Front. Immunol..

[3] Lin W., Li M., Li Y., Sun X., Li X., Yang F., Huang Y., Wang X. (2014). Bone marrow stromal cells promote neurite outgrowth of spinal motor neurons by means of neurotrophic factors in vitro. Neurol. Sci..

[4] Zilka N., Zilkova M., Kazmerova Z., Sarissky M., Cigankova V., Novak M. (2011). Mesenchymal stem cells rescue the Alzheimer’s disease cell model from cell death induced by misfolded truncated tau. Neuroscience.

[5] Zhao Y., Chen X., Wu Y., Wang Y., Li Y., Xiang C. (2018). Transplantation of Human Menstrual Blood-Derived Mesenchymal Stem Cells Alleviates Alzheimer’s Disease-Like Pathology in APP/PS1 Transgenic Mice. Front. Mol. Neurosci..

[6] Lee J.K., Jin H.K., Endo S., Schuchman E.H., Carter J.E., Bae J.S. (2010). Intracerebral transplantation of bone marrow-derived mesenchymal stem cells reduces amyloid-beta deposition and rescues memory deficits in Alzheimer’s disease mice by modulation of immune responses. Stem Cells.

[7] Babaei P., Soltani Tehrani B., Alizadeh A. (2012). Transplanted bone marrow mesenchymal stem cells improve memory in rat models of Alzheimer’s disease. Stem Cells Int..

[8] Scuteri A., Cassetti A., Tredici G. (2006). Adult mesenchymal stem cells rescue dorsal root ganglia neurons from dying. Brain Res..

[9] Monfrini M., Ravasi M., Maggioni D., Donzelli E., Tredici G., Cavaletti G., Scuteri A. (2018). Comparing the different response of PNS and CNS injured neurons to mesenchymal stem cell treatment. Mol. Cell Neurosci..

[10] Pittenger M.F., Discher D.E., Péault B.M., Phinney D.G., Hare J.M., Caplan A.I. (2019). Mesenchymal stem cell perspective: Cell biology to clinical progress. NPJ Regen. Med..

[11] Murray L.M.A., Krasnodembskaya A.D. (2019). Concise Review: Intercellular Communication Via Organelle Transfer in the Biology and Therapeutic Applications of Stem Cells. Stem Cells.

[12] Nawaz M., Fatima F. (2017). Extracellular Vesicles, Tunneling Nanotubes, and Cellular Interplay: Synergies and Missing Links. Front. Mol. Biosci..

[13] Nielsen M.S., Axelsen L.N., Sorgen P.L., Verma V., Delmar M., Holstein-Rathlou N.H. (2012). Gap junctions. Compr. Physiol..

[14] Carbone A., Zefferino R., Beccia E., Casavola V., Castellani S., Di Gioia S., Giannone V., Seia M., Angiolillo A., Colombo C. (2018). Gap Junctions Are Involved in the Rescue of CFTR-Dependent Chloride Efflux by Amniotic Mesenchymal Stem Cells in Coculture with Cystic Fibrosis CFBE41o- Cells. Stem Cells Int..

[15] Hirschi K.K., Burt J.M., Hirschi K.D., Dai C. (2003). Gap junction communication mediates transforming growth factor-beta activation and endothelial-induced mural cell differentiation. Circ. Res..

[16] Lemcke H., Gaebel R., Skorska A., Voronina N., Lux C.A., Petters J., Sasse S., Zarniko N., Steinhoff G., David R. (2017). Mechanisms of stem cell based cardiac repair-gap junctional signaling promotes the cardiac lineage specification of mesenchymal stem cells. Sci. Rep..

[17] Valiunas V., Doronin S., Valiuniene L., Potapova I., Zuckerman J., Walcott B., Robinson R.B., Rosen M.R., Brink P.R., Cohen I.S. (2004). Human mesenchymal stem cells make cardiac connexins and form functional gap junctions. J. Physiol..

[18] Jansens R.J.J., Tishchenko A., Favoreel H.W. (2020). Bridging the Gap: Virus Long-Distance Spread via Tunneling Nanotubes. J. Virol..

[19] Vignais M.L., Caicedo A., Brondello J.M., Jorgensen C. (2017). Cell Connections by Tunneling Nanotubes: Effects of Mitochondrial Trafficking on Target Cell Metabolism, Homeostasis, and Response to Therapy. Stem Cells Int..

[20] Doeppner T.R., Herz J., Görgens A., Schlechter J., Ludwig A.K., Radtke S., de Miroschedji K., Horn P.A., Giebel B., Hermann D.M. (2015). Extracellular Vesicles Improve Post-Stroke Neuroregeneration and Prevent Postischemic Immunosuppression. Stem Cells Transl. Med..

[21] Kim S.J., Moon G.J., Cho Y.H., Kang H.Y., Hyung N.K., Kim D., Lee J.H., Nam J.Y., Bang O.Y. (2012). Circulating mesenchymal stem cells microparticles in patients with cerebrovascular disease. PLoS ONE.

[22] Zhang J., Guan J., Niu X., Hu G., Guo S., Li Q., Xie Z., Zhang C., Wang Y. (2015). Exosomes released from human induced pluripotent stem cells-derived MSCs facilitate cutaneous wound healing by promoting collagen synthesis and angiogenesis. J. Transl. Med..

[23] Sartori-Rupp A., Cordero Cervantes D., Pepe A., Gousset K., Delage E., Corroyer-Dulmont S., Schmitt C., Krijnse-Locker J., Zurzolo C. (2019). Correlative cryo-electron microscopy reveals the structure of TNTs in neuronal cells. Nat. Commun..

[24] O’Donnell J.J., Birukova A.A., Beyer E.C., Birukov K.G. (2014). Gap junction protein connexin43 exacerbates lung vascular permeability. PLoS ONE.

[25] Mohammadalipour A., Dumbali S.P., Wenzel P.L. (2020). Mitochondrial Transfer and Regulators of Mesenchymal Stromal Cell Function and Therapeutic Efficacy. Front. Cell Dev. Biol..

[26] Raposo G., Stoorvogel W. (2013). Extracellular vesicles: Exosomes, microvesicles, and friends. J. Cell Biol..

[27] Aryani A., Denecke B. (2016). Exosomes as a Nanodelivery System: A Key to the Future of Neuromedicine?. Mol. Neurobiol..

[28] Yasuhara T., Kawauchi S., Kin K., Morimoto J., Kameda M., Sasaki T., Bonsack B., Kingsbury C., Tajiri N., Borlongan C.V. (2020). Cell therapy for central nervous system disorders: Current obstacles to progress. CNS Neurosci. Ther..

[29] Fan X.L., Zhang Y., Li X., Fu Q.L. (2020). Mechanisms underlying the protective effects of mesenchymal stem cell-based therapy. Cell Mol. Life Sci..

[30] Joyce N., Annett G., Wirthlin L., Olson S., Bauer G., Nolta J.A. (2010). Mesenchymal stem cells for the treatment of neurodegenerative disease. Regen. Med..

[31] Söhl G., Maxeiner S., Willecke K. (2005). Expression and functions of neuronal gap junctions. Nat. Rev. Neurosci..

[32] Rouach N., Glowinski J., Giaume C. (2000). Activity-dependent neuronal control of gap-junctional communication in astrocytes. J. Cell Biol..

[33] Charvériat M., Naus C.C., Leybaert L., Sáez J.C., Giaume C. (2017). Connexin-Dependent Neuroglial Networking as a New Therapeutic Target. Front. Cell Neurosci..

[34] Dilger N., Neehus A.L., Grieger K., Hoffmann A., Menssen M., Ngezahayo A. (2020). Gap Junction Dependent Cell Communication Is Modulated During Transdifferentiation of Mesenchymal Stem/Stromal Cells Towards Neuron-Like Cells. Front. Cell Dev. Biol..

[35] Sinclair K.A., Yerkovich S.T., Hopkins P.M., Chambers D.C. (2016). Characterization of intercellular communication and mitochondrial donation by mesenchymal stromal cells derived from the human lung. Stem Cell Res. Ther..

[36] Jolly C., Sattentau Q.J. (2004). Retroviral spread by induction of virological synapses. Traffic.

[37] Dieriks B.V., Park T.I., Fourie C., Faull R.L., Dragunow M., Curtis M.A. (2017). α-synuclein transfer through tunneling nanotubes occurs in SH-SY5Y cells and primary brain pericytes from Parkinson’s disease patients. Sci. Rep..

[38] Plotnikov E.Y., Khryapenkova T.G., Vasileva A.K., Marey M.V., Galkina S.I., Isaev N.K., Sheval E.V., Polyakov V.Y., Sukhikh G.T., Zorov D.B. (2008). Cell-to-cell cross-talk between mesenchymal stem cells and cardiomyocytes in co-culture. J. Cell. Mol. Med..

[39] Hsu M.J., Karkossa I., Schäfer I., Christ M., Kühne H., Schubert K., Rolle-Kampczyk U.E., Kalkhof S., Nickel S., Seibel P. (2020). Mitochondrial Transfer by Human Mesenchymal Stromal Cells Ameliorates Hepatocyte Lipid Load in a Mouse Model of NASH. Biomedicines.

[40] Verri M., Pastoris O., Dossena M., Aquilani R., Guerriero F., Cuzzoni G., Venturini L., Ricevuti G., Bongiorno A.I. (2012). Mitochondrial alterations, oxidative stress and neuroinflammation in Alzheimer’s disease. Int. J. Immunopathol. Pharmacol..

[41] Zhang Y., Yu Z., Jiang D., Liang X., Liao S., Zhang Z., Yue W., Li X., Chiu S.M., Chai Y.H. (2016). iPSC-MSCs with High Intrinsic MIRO1 and Sensitivity to TNF-α Yield Efficacious Mitochondrial Transfer to Rescue Anthracycline-Induced Cardiomyopathy. Stem Cell Rep..

[42] Wang Y., Cui J., Sun X., Zhang Y. (2011). Tunneling-nanotube development in astrocytes depends on p53 activation. Cell Death Differ..

[43] Mahrouf-Yorgov M., Augeul L., Da Silva C.C., Jourdan M., Rigolet M., Manin S., Ferrera R., Ovize M., Henry A., Guguin A. (2017). Mesenchymal stem cells sense mitochondria released from damaged cells as danger signals to activate their rescue properties. Cell Death Differ..

[44] Islam M.N., Das S.R., Emin M.T., Wei M., Sun L., Westphalen K., Rowlands D.J., Quadri S.K., Bhattacharya S., Bhattacharya J. (2012). Mitochondrial transfer from bone-marrow-derived stromal cells to pulmonary alveoli protects against acute lung injury. Nat. Med..

[45] Ahmad T., Mukherjee S., Pattnaik B., Kumar M., Singh S., Rehman R., Tiwari B.K., Jha K.A., Barhanpurkar A.P., Wani M.R. (2014). Miro1 regulates intercellular mitochondrial transport & enhances mesenchymal stem cell rescue efficacy. EMBO J..

[46] Galluzzi L., Kepp O., Kroemer G. (2012). Mitochondria: Master regulators of danger signalling. Nat. Rev. Mol. Cell Biol..

[47] Bruno S., Tapparo M., Collino F., Chiabotto G., Deregibus M.C., Soares Lindoso R., Neri F., Kholia S., Giunti S., Wen S. (2017). Renal Regenerative Potential of Different Extracellular Vesicle Populations Derived from Bone Marrow Mesenchymal Stromal Cells. Tissue Eng. Part A.

[48] Gonzalez-King H., García N.A., Ontoria-Oviedo I., Ciria M., Montero J.A., Sepúlveda P. (2017). Hypoxia Inducible Factor-1α Potentiates Jagged 1-Mediated Angiogenesis by Mesenchymal Stem Cell-Derived Exosomes. Stem Cells.

[49] Cui G.H., Wu J., Mou F.F., Xie W.H., Wang F.B., Wang Q.L., Fang J., Xu Y.W., Dong Y.R., Liu J.R. (2018). Exosomes derived from hypoxia-preconditioned mesenchymal stromal cells ameliorate cognitive decline by rescuing synaptic dysfunction and regulating inflammatory responses in APP/PS1 mice. FASEB J..

[50] Tao S.C., Yuan T., Zhang Y.L., Yin W.J., Guo S.C., Zhang C.Q. (2017). Exosomes derived from miR-140-5p-overexpressing human synovial mesenchymal stem cells enhance cartilage tissue regeneration and prevent osteoarthritis of the knee in a rat model. Theranostics.

[51] Eirin A., Zhu X.Y., Puranik A.S., Woollard J.R., Tang H., Dasari S., Lerman A., van Wijnen A.J., Lerman L.O. (2016). Comparative proteomic analysis of extracellular vesicles isolated from porcine adipose tissue-derived mesenchymal stem/stromal cells. Sci. Rep..

[52] Hu C., Li L. (2018). Preconditioning influences mesenchymal stem cell properties in vitro and in vivo. J. Cell. Mol. Med..

[53] Ferreira J.R., Teixeira G.Q., Santos S.G., Barbosa M.A., Almeida-Porada G., Gonçalves R.M. (2018). Mesenchymal Stromal Cell Secretome: Influencing Therapeutic Potential by Cellular Pre-conditioning. Front. Immunol..

[54] Chen Y.T., Tsai M.J., Hsieh N., Lo M.J., Lee M.J., Cheng H., Huang W.C. (2019). The superiority of conditioned medium derived from rapidly expanded mesenchymal stem cells for neural repair. Stem Cell Res. Ther..

[55] Scuteri A., Ravasi M., Monfrini M., Milano A., D’Amico G., Miloso M., Tredici G. (2015). Human Mesenchymal Stem Cells Protect Dorsal Root Ganglia from the Neurotoxic Effect of Cisplatin. Anticancer Res..

[56] Wood C.R., Juárez E.H., Ferrini F., Myint P., Innes J., Lossi L., Merighi A., Johnson W.E.B. (2021). Mesenchymal stem cell conditioned medium increases glial reactivity and decreases neuronal survival in spinal cord slice cultures. Biochem. Biophys. Rep..

