# Supplementary material for: Making Connections: Mesenchymal Stem Cells Manifold Ways to Interact with Neurons
Source: Int J Mol Sci. 2022 May 21;23(10):5791. doi: 10.3390/ijms23105791 (PMC9146463; doi:10.3390/ijms23105791)
Supplement: Supplementary file 1 [file ijms-23-05791-s001.zip › ijms-1686887-supplementary.pdf]

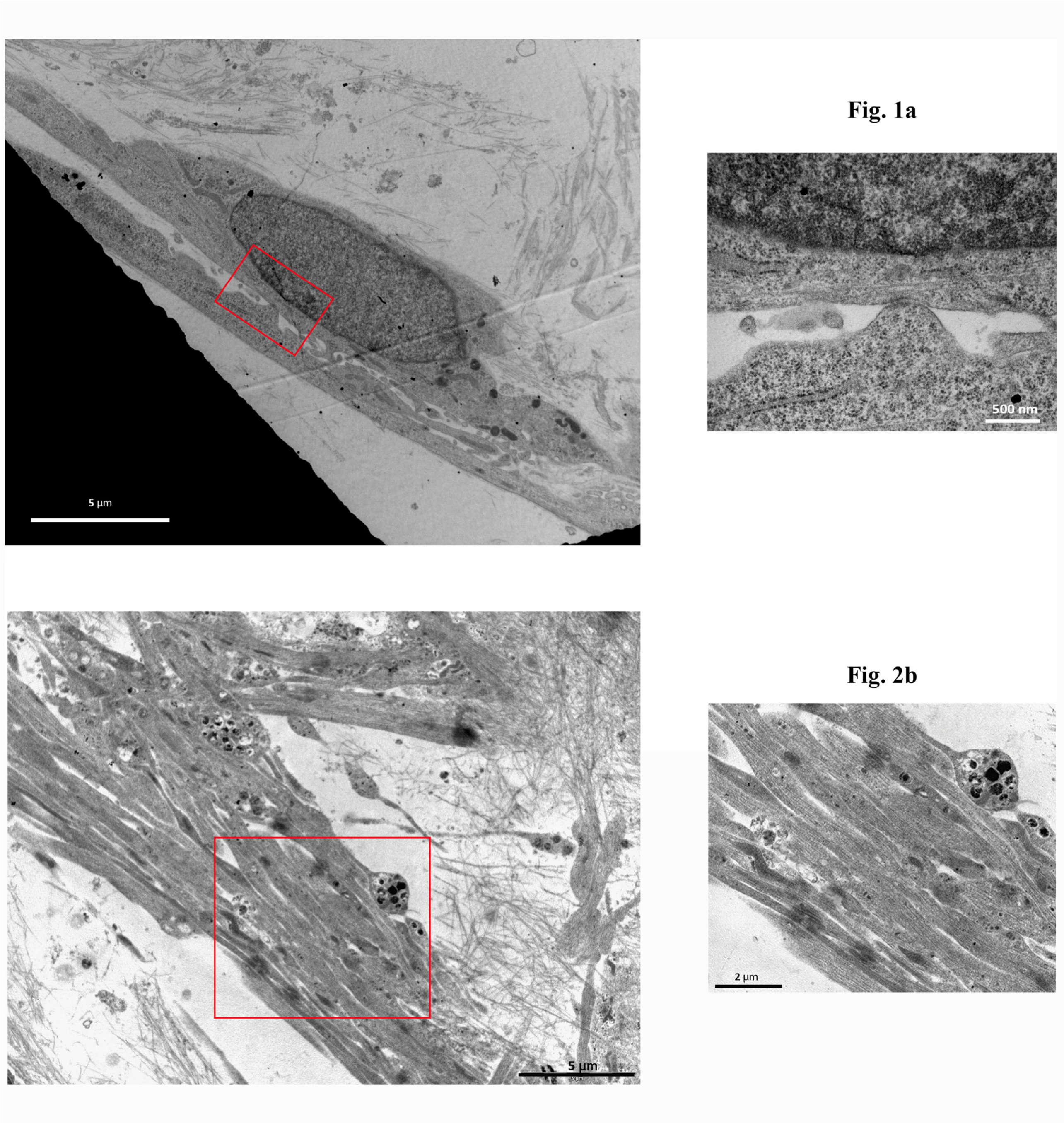

Figure S1. Low magnification images corresponding respectively to Fig. 1a (top) and Fig. 2b (bottom).
